# Radioactive Iodine for the Treatment of Subclinical Thyrotoxicosis Grade 1 and 2: Outcome of up to 18-Year Follow Up

**DOI:** 10.3389/fendo.2022.843857

**Published:** 2022-03-09

**Authors:** Jason Phowira, Katherine L. Coffey, Peter H. Bartholomew, Nicholas Vennart, Matheus Moreira, Hannah Emerson, David Kennedy, Jolanta U. Weaver

**Affiliations:** ^1^ Translational and Clinical Research Institute, Newcastle University, Newcastle upon Tyne, United Kingdom; ^2^ Department of Diabetes and Endocrinology, Queen Elizabeth Hospital, Gateshead, United Kingdom; ^3^ School of Pharmacy, Newcastle University, Newcastle upon Tyne, United Kingdom; ^4^ Medical Physics Department, South Tyneside and Sunderland NHS Foundation Trust, Newcastle upon Tyne, United Kingdom

**Keywords:** iodine radioisotopes, radiopharmaceuticals, subclinical thyrotoxicosis, toxic multinodular goitre, toxic nodule, Graves’ disease, treatment outcome

## Abstract

**Background:**

Subclinical thyrotoxicosis (SCT) is associated with significant morbidity and mortality, specifically increased risk of atrial fibrillation and cardiovascular death. The management is ill-defined due to the scarcity of randomised controlled studies. Some clinicians recommend radioiodine (RAI) treatment however its long-term outcome is unknown. Therefore, further data is needed to provide robust evidence-based guidelines.

**Methods:**

A prospective, single-protocol analysis of the outcome of SCT patients (Grade 1; 0.1-0.4 mIU/L and Grade 2; <0.1 mIU/L) treated with mean dose of 427 MBq of I^131^, followed up for up to 18 years. Thyroid function tests were measured at 4-6 weeks, 3-, 6-, and 12-months post-RAI, and annually thereafter. Cure was defined as achieving a euthyroid/hypothyroid state.

**Results:**

Seventy-eight patients with a median age of 68 years (range 36-84) and varying aetiology [55 toxic multinodular goitre (TMNG), 10 toxic nodule (TN) and 13 Graves’ disease (GD)] were followed up for a median period of 7.5 years (range 1-18). The cure rate was 100%. The rates of hypothyroidism in TMNG, TN and GD were 23.6%, 30% and 38.5% respectively. The median time to hypothyroidism was 6 and 12 months in GD and TMNG/TN respectively. No differences in outcome between Grade 1 versus Grade 2 were observed.

**Conclusion:**

RAI using single mean dose of 427 MBq is effective and safe, irrespective of aetiology or grade of TSH suppression. GD patients become hypothyroid within the first year, whilst TMNG/TN for up to 9-years. Thus after 12 months of follow up, annual thyroid function monitoring is advised.

## Introduction

Subclinical thyrotoxicosis (SCT) was first recognized as a clinical entity in the late 1980s. Since then the management of SCT has been widely debated (1).

SCT is the mildest form of thyrotoxicosis and is defined by low or undetectable serum thyroid-stimulating hormone (TSH) levels, with free thyroxine (FT4) and free triiodothyronine (FT3) levels within the reference range (2). The causes of SCT mirror those of overt thyrotoxicosis; including toxic multinodular goitre (TMNG), toxic nodule (TN) and Graves’ disease (GD). Patients with SCT can be further classified depending on the severity of their TSH suppression. A low but detectable TSH level (between 0.1-0.39 mIU/L) is deemed mildly suppressed (Grade 1) in comparison to a TSH value of <0.1 mIU/L (Grade 2) (3). In the UK, the prevalence of SCT was found to be 2.1% in a cross-sectional screening survey of 5,950 adults aged over 65 years (4).

In recent years, an increasing body of evidence has linked SCT to significant morbidity, and excess mortality. Selmer et al. described a dose-response relationship between TSH levels and an increased risk of atrial fibrillation (AF) (5). Most notably, patients with SCT and a TSH of 0.1-0.2 mIU/L had a 14% increased risk of AF compared to euthyroid patients, whereas those with G2 SCT had a 29% increased risk. SCT has also been demonstrated to cause a net increase in resorption of cortical bone, lead to reduced bone mineral density and the development of osteoporosis and/or pathological fractures (6). In addition, an earlier cohort study conducted by Parle and co-workers found a link between low TSH levels and increased all-cause mortality, in particular mortality due to cardiovascular diseases (4). However, it is also worth noting that, the presence on autonomously functioning thyroid nodule (AFTN) rather than a low serum TSH may represent a more effective and reliable approach in detecting AF, even when the patients are euthyroid (7, 8).

The absence of large, controlled intervention studies means there is no high-quality evidence on which to base treatment recommendations. As a result, guidelines produced by various professional groups illustrate uniform uncertainty. A brief synopsis of these guidelines is included in **Table 1**. In particular, the treatment of GD patients or Grade 1 SCT is poorly defined. As a result, individual endocrinologists are left to determine their own treatment policies, in the absence a good clinical evidence-base.

**Table 1:** Synopsis of SCT treatment guidelines produced by professional bodies.

Professional body	Publication Date	Recommendation
American College of Physicians	1998	No agreement on benefits of treating SCT
ATA, American Association of Clinical Endocrinologists (AACE) Endocrine Society Consensus Conference	2011	Strongly consider treatment in all individuals ≥65 years of age when TSH is <0.1 mIU/L; in postmenopausal women who are not on oestrogens or bisphosphonates; patients with cardiac risk factors, heart disease or osteoporosis; and symptomatic patients
European Thyroid Association	2015	Treat patients ≥ 65 years with TSH below <0.1 mIU/L. Consider treatment in patients ≥65 years with TSH levels 0.1–0.39 mIU/L because of increased risk of AFib. Treatment may be reasonable in symptomatic patients <65 with TSH persistently below <0.1 mIU/L especially if symptomatic or in the presence of underlying risk factors.
American Thyroid Association (ATA)	2016	Over 65 years: treat patients over 65 with TSH levels persistently less than 0.1 mIU/L. Consider Treatment If TSH levels are 0.1 to 0.4 mIU/L. Under 65 years: Treat patients with TSH levels less than 0.1 mIU/L with co-morbid heart disease, osteoporosis or post-menopausal (without oestrogen or bisophosphonate therapy). Consider treatment in; asymptomatic patients with TSH levels less than 0.1 mIU/L, symptomatic of hyperthyroidism and patients with TSH levels of 0.1 to 0.4 mIU/L co-morbid heart disease, osteoporosis, post-menopausal without oestrogen or bisophosphonate therapy
NICE	2019	Consider seeking specialist advice on managing subclinical hyperthyroidism in adults if they have: two TSH readings <0.1 mIU/L at least 3 months apart and evidence of thyroid disease (for example, a goitre, positive thyroid antibodies or symptoms of thyrotoxicosis). People between the ages of 65 and 80 are likely to be those who may benefit from treatment most. Under 65s are likely to be low risk from SCT therefore initiate treatment when condition becomes clinical. In people over the age of 80 there is a high chance that a low TSH is related to advanced age or co-morbidities.

Some endocrinologists advocate the use of radioiodine (RAI) therapy for treating SCT. RAI is preferable over antithyroid drug therapy (ATDs) in most cases of SCT due to its ability to induce permanent cure after a single dose of I^131^, and relative lack of adverse effects. One-time treatment with RAI has been found to be more cost-effective than long periods of ATD therapy (9).

Whilst RAI appears to be a promising treatment for SCT, the evidence-base for its use is limited. Currently only eight non-randomised studies have investigated the use of RAI for SCT. Across the studies the cure rate of RAI was high, with five studies reporting 100% of patients became euthyroid, and the lowest reported cure rate being 83.3%. However, the mean number of patients included across these studies was only 24, with one study including just 6 patients. The longest duration of patient follow-up was 3 years, with one study spanning only 9 months. Only one study was a randomised controlled trial (10). Furthermore, only a single study involved patients with GD and no studies described results depending on the baseline level of TSH suppression. As a result, there is unmet need for clinical information on SCT management. The present study aimed to analyse the long-term outcome of RAI therapy in SCT patients of varying grades and aetiologies.

## Materials and Methods

Since year 2003, a prospective audit of treatment outcome in 78 patients with SCT treated with radioiodine at a general hospital in the UK was carried out.

Patients with suspected SCT were mainly referred by primary care physicians. The diagnosis of SCT was confirmed by the presence of successive abnormal serum TSH results, documented at least 3 months apart with TSH levels, less than 0.4 mIU/L, and both free triiodothyronine (FT3) and free thyroxine (FT4) levels within reference range. Patients who showed normalisation of thyroid function or progression to overt thyrotoxicosis on repeat testing were excluded from the audit. The diagnosis of GD, TMNG or TN was established on the history, clinical examination and presence of either elevated TSH receptor antibodies (TRAb) or thyroid isotope uptake or both. Patients with previous history of thyroid surgery, treatment with thyroxine or anti-thyroid drug therapy were excluded.

All patients were offered choice of RAI therapy, anti-thyroid drugs (ATD) or a ‘wait and watch’ policy. Patients who consented and underwent RAI therapy were included in our audit. RAI was administered on an outpatient basis at prescribed dose of 400 MBq of I^131^. ARSAC guidance recommends that the activity administered is within 10% of the reference activity, thus patients prescribed with 400 MBq receive between 360-440 MBq. The mean dose of RAI received by our patients was 427 MBq due to the manufacturing process of radioisotopes.

Following treatment, all patients attended for regular blood monitoring, specifically TSH and FT4/FT3 levels at 4-6 weeks, 3, 6, 9 and 12-months post-RAI treatment and annually thereafter until up to 18 years post-treatment. Patients remained under the care of the single endocrinologist until they became euthyroid on no thyroxine therapy or persistently hypothyroid, at which time they were treated if required and discharged back to their General Practitioner’s care for further follow up.

The clinical information, adverse events including death were obtained from the hospital records.

The cure was defined as patient achieving either euthyroid or hypothyroid state following a single dosage of RAI therapy, and a ‘treatment failure’ was defined as (1) patient who remained subclinically thyrotoxic at their final follow-up or (2) patient who required repeated dose of RAI.

Serum TRAb, TSH, FT4 and FT3 levels were measured by an electro-chemiluminescent immunoassay (ECLIA) using the Roche immunoassays. The normal ranges were as follows: TRAb <1.8 U/L, TSH, 0.4-4.0 mIU/L; FT3, 3.5-6.8 pmol/L; FT4, 9.0-25.0 pmol/L.

### Statistical Analysis

The results were expressed as mean ± standard deviation or as stated otherwise. Chi-square test was performed to compare the prevalence of euthyroid or hypothyroid between the three aetiologies. Kruskall-Wallis test was performed to compare the time to euthyroid or hypothyroid states between the three conditions. Kaplan-Meier analysis was used to generate survival curves to describe the proportion of patients who became euthyroid or hypothyroid according to the aetiology of SCT (11). Significance was accepted when p<0.05. The statistical analyses were performed using GraphPad Prism 9.0 (GraphPad Software, San Diego, USA). All analyses were performed on anonymised data sets.

### Ethical Approval

This audit was registered with the Clinical Audit Department at local hospital. Ethical approval was deemed not necessary for the audit of existing patient data. However, all activity was in accordance with the 1998 Data Protection Act, the NHS Confidentiality Code of Practice (2003) and the Caldicott Principles (1997).

## Results

Data from 78 patients (71 females) were analysed. The baseline demographic and laboratory characteristics are outlined in **Table 2**. The breakdown of diagnosis by the degree of suppression (G1 versus G2) is provided in **Table 3**.

**Table 2:** Baseline characteristics of patients with SCT.

Characteristic	All patients (n=78)	GD (n=13)	TMNG (n=55)	TN (n=10)
Median Age (Range)	68 (36-84)	63 (57-82)	67 (36-84)	60.5 (50-76)
Sex Male	7 (9.0%)	1 (7.7%)	4 (7.3%)	2 (20%)
Female	71 (91.0%)	12 (92.3%)	51 (92.7%)	8 (80%)
Median TSH (mIU/L)	0.11	0.11	0.13	0.05
TSH Range (mIU/L)	<0.01-0.38	<0.01-0.29	<0.01-0.34	<0.02-0.38
Grade 1 Suppression (0.10-0.39mIU/L)	22 (28.2%)	2 (15.4%)	19 (34.5%)	1 (10%)
Grade 2 Suppression (<0.10mIU/L)	56 (71.8%)	11 (84.6%)	36 (65.5%)	9 (90%)
Mean FT4 ± SD	16.9 ± 2.6	17.0 ± 2.6	16.7 ± 2.5	17.7 ± 2.9
FT4 Range (pmol/L)	11.9-23.8	12.7-22.0	11.9-23.8	13.2-23.7
Mean FT3 ± SD	5.6 ± 0.9	5.4 ± 0.6	5.5 ± 0.7	5.8 ± 0.79
FT3 Range (pmol/L)	4.2-6.8	4.2-6.8	4.2-6.8	4.5-6.8
Mean dose of RAI (MBq)	427	421	429	423

Data are presented as median (range) or mean ± SD. FT3, free triiodothyronine; FT4, free thyroxine; GD, Graves’ disease; RAI, radioiodine; TMNG, toxic multinodular goitre; TN, toxic nodule; TSH, thyroid-stimulating hormone.

**Table 3:** Severity of TSH Suppression, classified by SCT aetiology.

Aetiology	Grade 1 TSH Suppression (0.1-0.39mIU/L) n=22	Grade 2 TSH Suppression (<0.1mIU/L) n=56
TMNG	19 (86.4%)	36 (64.3%)
TN	1 (4.5%)	9 (16.1%)
GD	2 (9.1%)	11 (19.6%)

Data are presented as n (%). GD, Graves’ disease; TMNG, toxic multinodular goitre; TN, toxic nodule; TSH, thyroid-stimulating hormone.

### Cure Rate

At final follow-up of all patients, 73.1% were euthyroid and 26.9% were hypothyroid, giving the cure rate of RAI after 18 years to be 100% with a failure rate of 0%. The prevalence of hypothyroidism in patients with TN, TMNG and GD after 18-years was 30%, 23.6%, and 38.5% respectively (**Table 4**). No differences in outcome between Grade 1 versus Grade 2 were observed (**Table 5**).

**Table 4:** Outcome of RAI Therapy in all patients, classified by SCT aetiology.

Outcome of RAI	All patients (n=78)	GD (n=13)	TMNG (n=55)	TN (n=10)
Euthyroid	57 (73.1%)	8 (61.5%)	42 (76.4%)	7 (70%)
Hypothyroid (SCH or overt)	21 (26.9%)	5 (38.5%)	13 (23.6%)	3 (30%)
Treatment Failure (persistent SCT)	0 (0%)	0 (0%)	0 (0%)	0 (0%)
**Cure Rate**	**100%**	**100%**	**100%**	**100%**

Data are presented as n (%). GD, Graves’ disease; RAI, radioiodine; SCH, Subclinical hypothyroidism; SCT, Subclinical thyrotoxicosis; TMNG, toxic multinodular goiter; TN, toxic nodule. A euthyroid or hypothyroid outcome at the end of the follow up period is classified as treatment success.

**Table 5:** Post-RAI outcome classified by severity of TSH suppression.

Outcome of RAI	Grade 1 TSH Suppression (0.1-0.39mIU/L) n=22	Grade 2 TSH Suppression (<0.1mIU/L) n=56
Euthyroid	17 (77.3%)	41 (73.2%)
Hypothyroid (SCH or Overt)	5 (22.7%)	15 (26.8%)
Treatment Failure (persistent SCT)	0 (0%)	0 (0%)
**Cure Rate**	**22 (100%)**	**56 (100%)**

Data are presented as n (%). RAI, radioiodine; SCH, Subclinical hypothyroidism; SCT, Subclinical thyrotoxicosis; TSH, thyroid-stimulating hormone.

### Euthyroidism and Hypothyroidism

The median time to euthyroidism was 3 months (range 1.5 months to 2 years). For the entire cohort, the vast majority (93.5%) of patients who achieved euthyroidism had done so by 12 months. Most patients with TMNG and all TN patients achieved euthyroidism by 12 months, whilst with GD by 6 months. SCT persisted for 2 years in one GD patient and two TMNG patients. Statistical analyses revealed that the underlying aetiology of SCT had no statistically significant effect on the prevalence (p=0.54) and time to achieving euthyroidism (p=0.68).

The rate of progression to hypothyroidism varied depending on the aetiology of SCT. The median time to hypothyroidism in TMNG and TN patients was 12 months (range 1.5 months to 9 years), whilst for GD was 6 months (range 3-12 months). All GD patients who became hypothyroid did so within the first 12 months, whilst TMNG patients were still becoming hypothyroid up to 9 years after RAI. Statistical analyses revealed that the underlying aetiology of SCT had no statistically significant effect on the prevalence (p=0.54) and time to achieving hypothyroidism (p=0.22).

**Figure 1:**
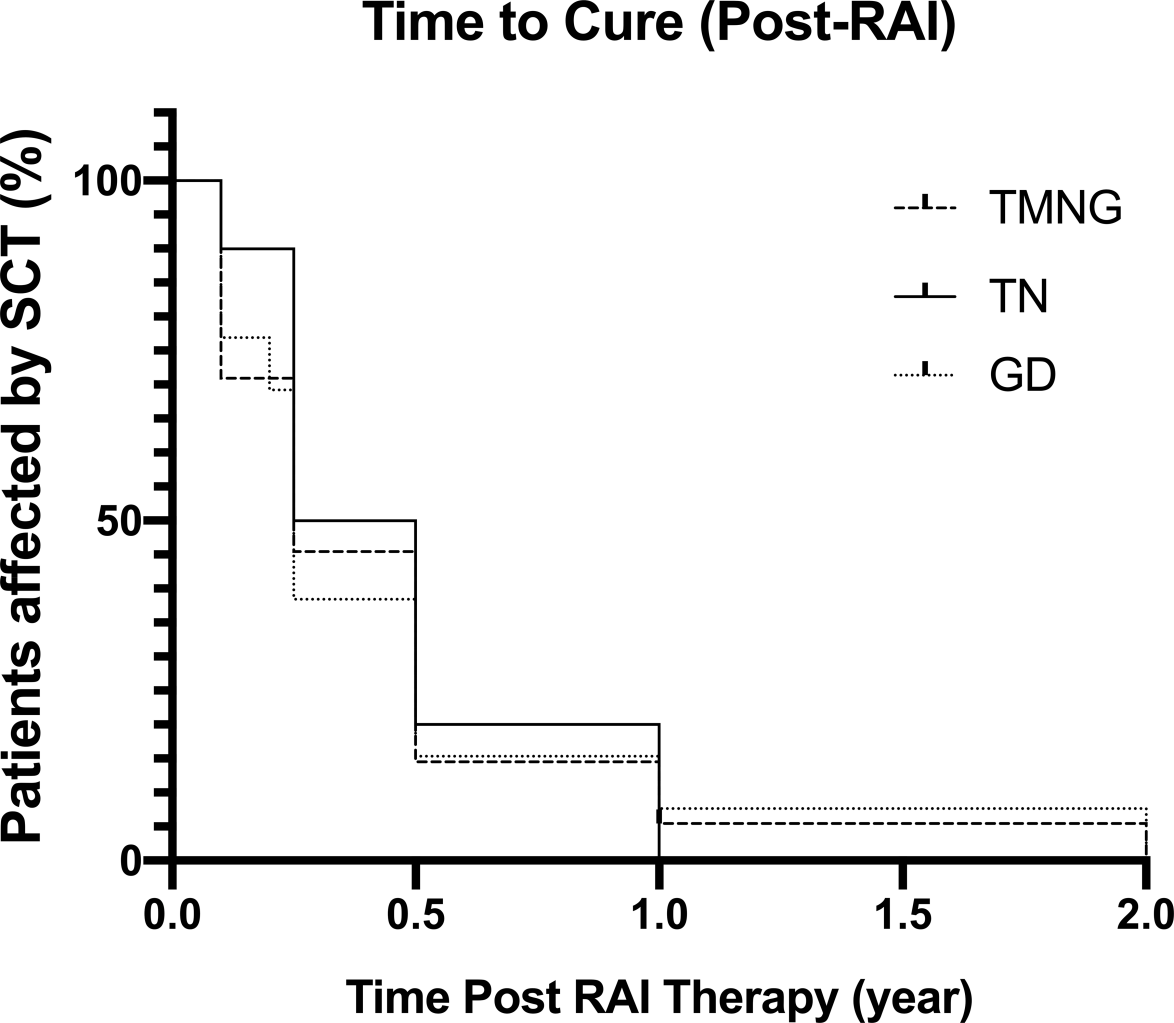
Kaplan-Meier Graph showing time taken post-RAI to reach euthyroid or hypothyroid status, defined as cure.

Kaplan-Meier analysis in **Figure 1** represents the time needed for patients in achieving either euthyroid or hypothyroid state following a single dosage of RAI therapy.

#### Toxic Multinodular Goitre

At final follow-up, 76.4% of TMNG patients were euthyroid and 23.6% were hypothyroid. The prevalence of hypothyroidism in TMNG patients gradually increased with time, with one patient becoming hypothyroid 9-years post-radioiodine.

#### Toxic Nodule

At final follow-up, 70% of TN patients were euthyroid whilst 30% became hypo-thyroid. The prevalence of hypothyroidism gradually increased with time up to 3-years post-treatment. All patients with TN who became euthyroid did so within 12-months.

#### Graves’ Disease

In GD patients, at final follow-up, 61.5% were euthyroid and 38.5% were hypo-thyroid. All patients who were hypothyroid did so within 12-month of RAI therapy period.

**Figure 2:**
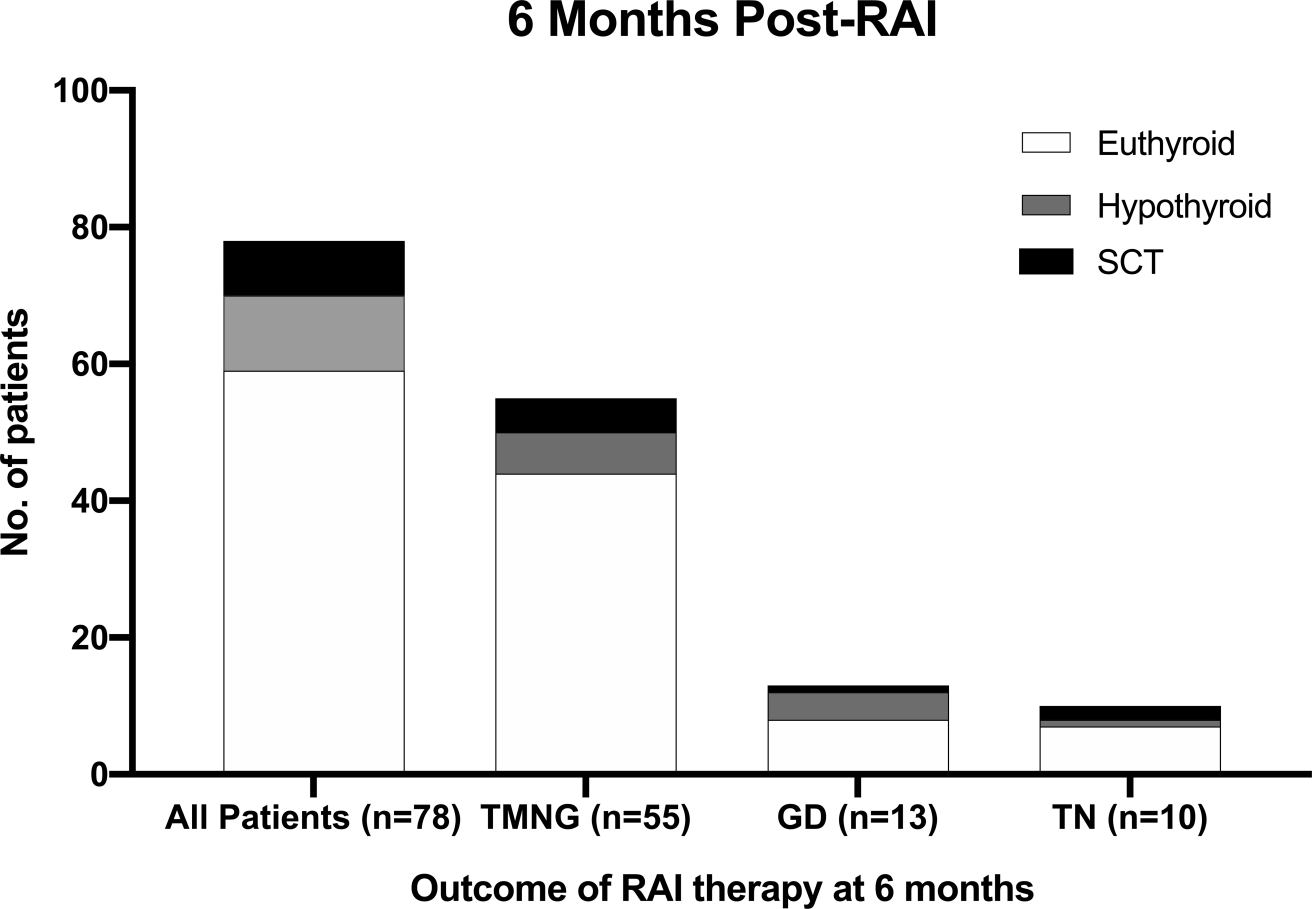
Outcome for SCT patients 6-month post-RAI therapy.

**Figure 3:**
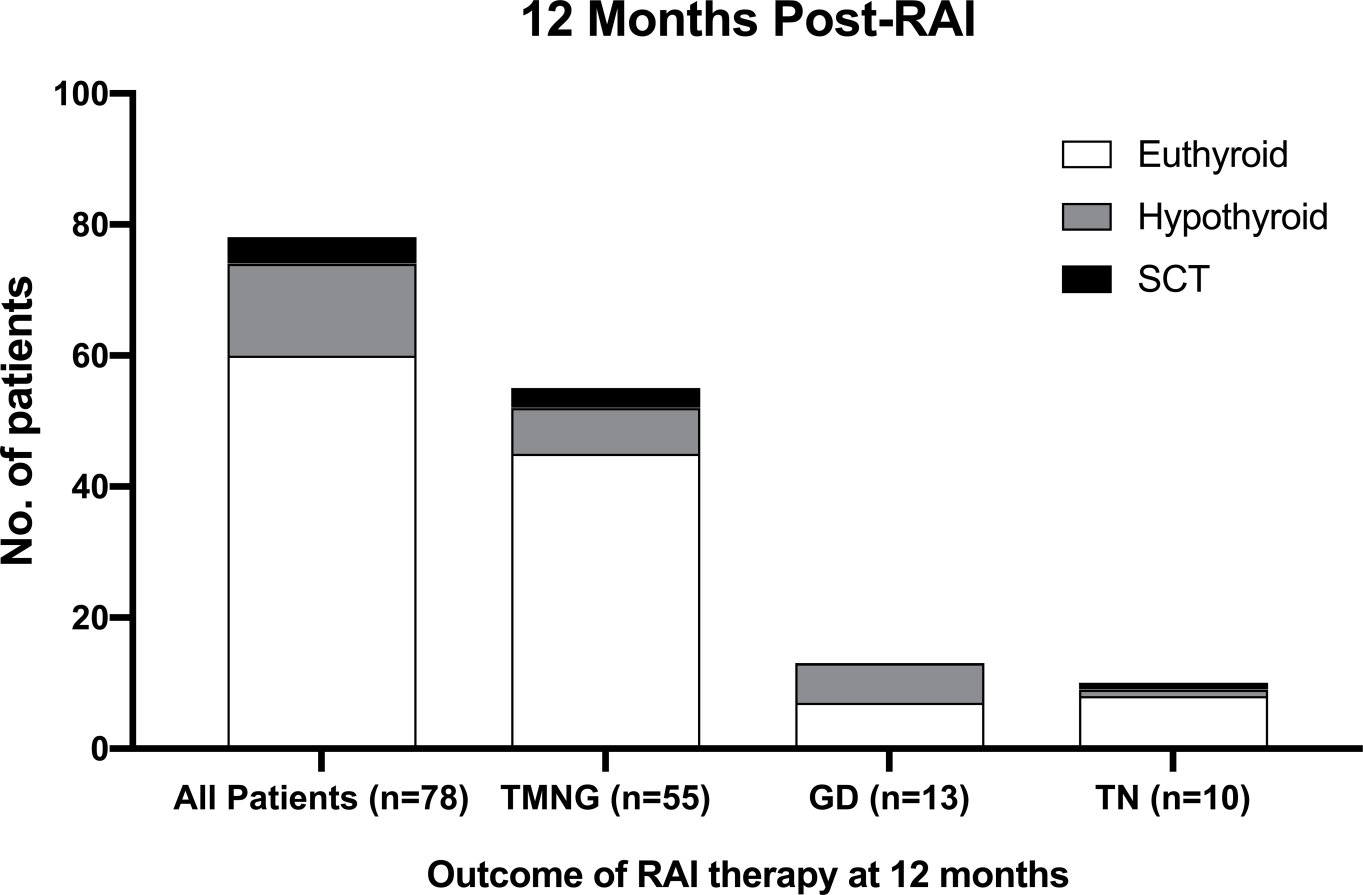
Outcome for SCT patients 12-month post-RAI therapy.

The thyroid function results of TMNG, TN, GD patients at 12 months and 3 years post-RAI are illustrated in **Figures 2** and **3**.

No cases of thyroiditis or thyroid orbitopathy were recorded, nor other adverse effects were noted for the entire follow-up period.

During the audit period, 27 cases (23 TMNG, 4 GD) of all-cause mortality were recorded (mean age at death 80.5 years). The mean age of death in the TMNG and GD patient groups were 80.4 and 81 years, respectively. There was no death recorded among the TN patient group.

When data after removing small number of patients with GD was reanalysed, the results did not differ significantly with respect to the patients’ treatment response, the prevalence of euthyroidism or hypothyroidism, as well as the rate to achieving euthyroidism or hypothyroidism between the three aetiologies.

## Discussion

To our knowledge, this is the largest and longest review of the outcome of radioiodine therapy, using single protocol employing single prescribed dose of 400 MBq of RAI in patients with SCT. The number of patients studied, and the duration of their follow-up is unparalleled. We analysed the data from 78 patients with SCT (TMNG, TN and GD) followed up for 18-years post-radioiodine treatment.

The most unexpected finding from our study was the similarity in outcomes for patients with Grade 1 in comparison to Grade 2 TSH suppression. Both the 2016 ATA and 2015 ETA guidelines for the treatment of SCT recommend therapy depending on the level of TSH suppression, specifically that Grade 2 suppression should be treated in most instances (3, 12). In our study, we found no significant difference in the outcome in patients with Grade 1 versus Grade 2 SCT. However, the comparison for GD group was not meaningful statistically as there were only 2 GD patients Grade 1 versus 11 with Grade 2. Both professional bodies recommend caution in treating patients with Grade 1 suppression due to the increased risk of the TSH suppression only being transient. It has therefore been argued that treatment might not be necessary as the condition will spontaneously resolve. Our protocol protected against the possibility of SCT being transient by ensuring TSH was suppressed on at least two occasions, at a minimum of 6 months apart. As we found no significant difference in the cure rate or the prevalence of hypothyroidism in patients with Grade 1 and Grade 2 suppression, our findings suggest that considering TSH suppression is permanent, there is no difference in outcome of RAI therapy irrespective of the baseline degree of TSH suppression.

Furthermore, no group has compared outcome of RAI therapy for all three disease states within the same protocol. In doing so we have been able to demonstrate differences in the outcome of treatment depending on the underlying cause of SCT.

### Cure Rate

In our investigations, radioiodine was effective in curing SCT in 100% of all cases. Whilst research describing outcomes post-RAI treatment is limited in number, two existing studies have reported similar cure rates (13, 14). Rosario et al. reported a cure rate of 83.3% in 36 women with SCT treated with RAI. Boj-Carceller et al. reported RAI was an effective cure for 94.1% of women in a study involving 17 subjects. Both these studies reported cure rates at just 12 months of follow-up. A further two studies reported higher cure rates of 100% after two years (15, 16). However the number of subjects in these studies was considerably smaller than in ours: 16 and 44 patients respectively, Similarly, the length of these studies was much shorter than ours, involving just two years of follow-up. We found the outcome of radioiodine administration could change in a patient for up to nine years post-therapy. As a result, the findings of studies shorter than nine years in duration may only be preliminary and the outcome subject to change with time.

Importantly, we found that the underlying cause of SCT may affect the response to RAI. Several studies have reported the cure rate of RAI for SCT due to TMNG or TN alone. Three studies reported similar findings to ours in patients with SCT due to TN (13, 14, 16). Both Faber et al. and Boj-Carceller et al. reported an 100% cure rate after 12-months, in just 16 and 17 patients respectively. Rosario et al. found a slightly lower cure rate of 83.3% in 36 women with TN, again 12-months after treatment (13). However this paper only investigated the effects of RAI in elderly patients over the age of 65-years, so is not directly comparable to our study. A further study by Faber et al. found the cure rate of RAI to be 100% in 6 patients with SCT due to TMNG (17), although results were reported just 276 days after RAI administration. Whilst the cure rates in these publications were similar to those in our study, this data has obvious limitations due to number of patients and length of follow-up period. Finally, the very recent trial by Azizi et al. demonstrated that RAI was effective in curing SCT in elderly patients, with cure rate of 95% (18).

In addition, our previous data on the outcome of RAI therapy in 201 patients with overt thyrotoxicosis using similar dose of RAI showed a cure rate of 93.5% (19). Whilst findings for overt thyrotoxicosis are not entirely applicable to SCT, there are similarities in outcome between the two disease states.

Existing treatment guidelines suggest that RAI should not be considered in GD patients due to the higher likelihood of spontaneous remission (3, 12). Our protocol began several years before the establishment of these guidelines, and therefore several GD patients were given RAI. In GD patient who are more likely to go into spontaneous remission, abnormal TSH levels were noted to span between 6 months and 5 years. Thus, we have considered the possibility of unnecessarily treating patients whose thyroid function could spontaneously normalise by testing TSH levels on several occasions, often over a time period of at least 12-months before proceeding to RAI.

### Euthyroidism

The underlying cause of SCT did not significantly affect the prevalence of euthyroidism (p=0.54) in our study. In regard to the time taken to become euthyroid following RAI therapy, for those patients that achieved euthyroidism, 93.5% had done so by 12-months post-therapy. SCT persisted for 2 years only in 1 GD and 2 TMNG patients. Our analysis demonstrated that the aetiology of SCT had no significant effect on the time taken to become euthyroid (p=0.68). The median time to euthyroidism in our study was 3 months, which is in line with the findings of existing publications, Mark et al. reported the median time to normalization of TSH to be 2.9 months (range 35-130 days) in 12 women with SCT due to TN (20). Moreover, Kaminski et al. reported a longer median time to euthyroidism of 6.9 months in 44 patients with SCT of non-autoimmune origin (21). However the exact activity of radioiodine administered to these patients was not disclosed, so direct comparison is not possible. Aziz et al. reported euthyroid rate of 34% after 5 years of follow up in patients treated with 15miCu of RAI (18).

Quantifying the time taken to reach euthyroidism has important implications for the management of patients after RAI. In our study, the maximum time to euthyroidism was 2 years in three patients, but otherwise with majority achieving euthyroidism after 12 months. It is thus reasonable to suggest that patients who have not become euthyroid/hypothyroid by this time point may not respond to treatment.

### Hypothyroidism

The overall rate of hypothyroidism amongst our patients was 26.9% after 18 years of follow up. In our audit, 38.5% of patients with GD become hypothyroid, in comparison with just 23.6% of patients with TMNG and 30% with TN. However, the underlying cause of SCT did not have a statistically significant effect on the prevalence of hypothyroidism (p=0.54) in our analysis.

With regards to the rate of progression to hypothyroidism, we found patients with GD became hypothyroid at a much faster rate than other subjects. The median time to hypothyroidism in patients with TMNG and TN was 12 months (range 1.5 months to 9 years), whilst for patients with GD the median time to hypothyroidism was just 6 months (range 3-12 months). Of those patients that did become hypothyroid, 100% of patients with GD did so within the first year of treatment. Patients with TMNG and TN became hypothyroid at a much slower rate, up to 9 years following treatment. Despite this finding, the aetiologies of SCT appeared to have no statistically significant effect on the rate of progression to hypothyroidism (p=0.22). This may be explained by the subclinical state of the diseases we were treating with RAI. The rates of hypothyroidism in our study are substantially higher than in some studies but not all of them. Rosario et al. detected hypothyroidism in only 2.7% of elderly females after 12 months (13). A second study involving 17 patients with TN reported a slightly higher prevalence of 5.9% (14). Kaminski et al. found only 6.8% of patients became hypothyroid after 36-months, although this study did not include any patients with SCT due to GD (15). Azizi et al. reported much high rate of hypothyroidism (66%) in the elderly group of patients with SCT (18). This high rate of hypothyroidism could be attributed to several factors including higher dose of RAI used by the investigators and higher number of patients presumed to have GD.

At 12-months post-RAI we reported the prevalence of hypothyroidism to be 19% across all patients. This value steadily increased to 26.9% at 18 years, illustrating the prevalence of hypothyroidism increases with time following RAI in SCT. As our audit included several GD patients with SCT, therefore we postulated that the inclusion of GD patients may contribute to the higher prevalence of hypothyroidism in our study.

Interestingly, the prevalence of hypothyroidism in patients with GD was much lower in current audit compared to our previous audit in overt thyrotoxicosis (38.5% vs 77.8%) (19). The quantity of RAI prescribed to patients was the same in both protocols, so this does not account for the discrepancy. The other reason may be the presence of Hashimoto’s thyroiditis, an autoimmune thyroid disease that may convert to GD and vice versa, contributed to this difference. This conversion is postulated to occur due to an imbalance between autoimmune mediated thyroid destruction and regeneration (22, 23). However, the prevalence of hypothyroidism in other conditions (TMNG and TN) was similar between the current audit and the previous one; TMNG (23.6% vs 29.1%) and TN (30% vs 30.7%) patients (24).

The ability to quantify the rate of progression to hypothyroidism following RAI therapy has important implications for patient safety. Increasing the frequency of thyroid function monitoring during the ‘danger time’ reduces the potential for hypothyroidism to go undetected for longer periods. This in turn reduces the risk of patients suffering from the complications of hypothyroidism and allows hormone replacement therapy to be started in a timely manner if necessary. The early detection of hypothyroidism following RAI therapy is particularly important for patients with GD as they are at increased risk of complications from thyroid-associated orbitopathy (24). During our study we reported no incidences of transient thyrotoxicosis, thyroid-associated orbitopathy or other adverse effects associated with radioiodine administration. Two previous studies reported transient thyrotoxicosis following treatment in 5.5% and 6.8% of patients retrospectively (13, 15).

The 2015 ETA guidelines for the treatment of SCT recommend periodic follow-up should be performed during the first year following RAI therapy, and then annually to assess normalization of thyroid function or the development of hypothyroidism. In our study we found 4-6 weeks, 3, 6 and 12 months to be sufficient intervals for monitoring TSH levels. There were notable differences in the incidence of hypothyroidism at each of these time points, suggesting that less frequent monitoring during the first year would miss hypothyroidism.

Consistently throughout our audit we have found that the underlying cause of SCT does not influence the final treatment outcome. Current guidelines also do not differentiate treatment policies based on the aetiology of SCT. The frequency of follow-up was the same for all patients in our audit, and our findings suggest that following RAI treatment, the frequency and duration of follow-up may be tailored depending on their aetiology. For instance, patients with GD should have an increased frequency of follow-up during the first year after treatment due to their high risk of hypothyroidism in this time. After 12-months, we found treatment outcome for patients with GD did not change, thus having an implication for the follow up. Conversely, patients with TMNG or TN should remain in active follow up for longer periods of time due to the possibility of them becoming hypothyroid as late as 9-years after therapy. Tailoring follow-up to the aetiology of SCT maximises patient safety whilst ensuring resources are used efficiently.

Our results show that RAI is very effective and safe in curing SCT regardless the aetiology and degree of TSH suppression. If implemented, it could reduce costs and improve patient outcomes. An alternative option to RAI, use of anti-thyroid drugs (ATD), is more costly than RAI, requires patients to adhere to regular visits to a physician, thyroid function monitoring and medication schedule which may be associated with adverse effects including agranulocytosis and hepatocellular disease (9).

The accurate outcome data allows endocrinologists to reliably inform their patients on the effect of treatment; whilst information on the onset of hypothyroidism will aid patients in making the decision regarding radioiodine treatment.

## Conclusion

RAI appears to be an effective first line treatment for SCT regardless of aetiology or grade of TSH suppression. Single dose protocol of prescribed 400 MBq of I^131^ offers high cure rate and is easy to be administered. Careful monitoring for the first year and annually afterwards as demonstrated in our protocol can identify early cases of hypothyroidism to commence thyroxine replacement therapy.

## Data Availability Statement

The original contributions presented in the study are included in the article/supplementary material. Further inquiries can be directed to the corresponding author.

## Ethics Statement

Ethical approval was not provided for this study on human participants because this audit was registered with the Clinical Audit Department at local hospital. Ethical approval was deemed not necessary for the audit of existing patient data. Written informed consent was not provided because of the retrospective nature of the study design.

## Author Contributions

Conceptualization, JU. methodology, JU. formal analysis, JP, KL, MM, HE, JU. investigation, DK. resources, PH, NV, DK, JU. data curation, PH, NV, JU. writing—original draft preparation, JP, KL, PH, NV, MM, DK, JU. writing—review and editing, JP, KL, PH, NV, MM, DK, JU. visualization, PH, DK, NV, JU. supervision, JU. project administration, JU. All authors have read and agreed to the published version of the manuscript.

## Conflict of Interest

The authors declare that the research was conducted in the absence of any commercial or financial relationships that could be construed as a potential conflict of interest.

## Publisher’s Note

All claims expressed in this article are solely those of the authors and do not necessarily represent those of their affiliated organizations, or those of the publisher, the editors and the reviewers. Any product that may be evaluated in this article, or claim that may be made by its manufacturer, is not guaranteed or endorsed by the publisher.
